# Spontaneously Resolving Periocular Erythema and Ciliary Madarosis Following Intra-arterial Chemotherapy for Retinoblastoma

**DOI:** 10.4103/0974-9233.65492

**Published:** 2010

**Authors:** Brian Marr, Pierre Y. Gobin, Ira J. Dunkel, Scott E. Brodie, David H. Abramson

**Affiliations:** Ophthalmic Oncology Service, Memorial Sloan-Kettering Cancer Center, New York, NY, USA; 1 Interventional Neuroradiology, Departments of Radiology, Neurosurgery and Neurology, Weill Cornell Medical College, New York, NY, USA; 2 Department of Pediatrics, Memorial Sloan-Kettering Cancer Center, New York, NY, USA; 3 Department of Ophthalmology, Mt. Sinai School of Medicine, New York, NY, USA

**Keywords:** Cancer, Eye, Intra-Arterial Chemotherapy, Melphalan, Retinoblastoma, Skin, Topotican

## Abstract

**Purpose and Design:**

To describe an unusual clinical finding seen in children undergoing intra-arterial chemotherapy for retinoblastoma.

**Materials and Methods:**

A retrospective review of 69 eyes of 63 patients receiving intra-arterial chemotherapy over a 3-year period. Charts and photographs of 69 consecutive cases were reviewed, and data were collected on patients with clinical evidence of a hyperemic cutaneous periocular abnormality following the procedure.

**Results:**

A blanching erythematous and edematous patch was noted in the periocular region in 16% (11 of 69) of the children who received intraarterial chemotherapy. The plaque extended into the region of the supertrochlear and medial marginal artery distribution on the ipsilateral side of the intra-arterial chemotherapy. All patches of erythema spontaneously resolved within 3 months following completion of the intra-arterial chemotherapy.

**Conclusion:**

Periocular erythema and swelling is a self-limited clinical finding associated with intra-arterial chemotherapy in a small number of patients.

## INTRODUCTION

Treatment using super selective intra-arterial chemotherapy has been used successfully to treat eyes with bilateral and unilateral retinoblastoma.^1^,^2^ Results on electroretinographic findings and efficacy have been described.^1^,^3^ Currently, swelling is the only adverse periocular effect reported in the English literature following such treatments.^1^ We describe an unusual clinical finding seen following intra-arterial chemotherapy.

## MATERIALS AND METHODS

**Figure 1:**
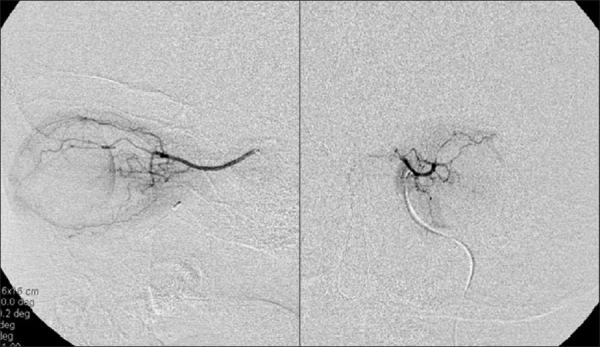
Ophthalmic artery angiogram of the left orbit, lateral, and posterior/anterior views

After an institutional review board approval, a retrospective review of 69 eyes receiving intra-arterial chemotherapy was performed on 63 consecutive patients beginning in May of 2006. The same physician preformed all procedures using the same technique (YPG)^1^ [Figure 1]. Charts and photographs were reviewed for patients having an associated hyperemic cutaneous periocular abnormality following chemosurgery; demographic and procedure data were collected.

## RESULTS

**Figure 2:**
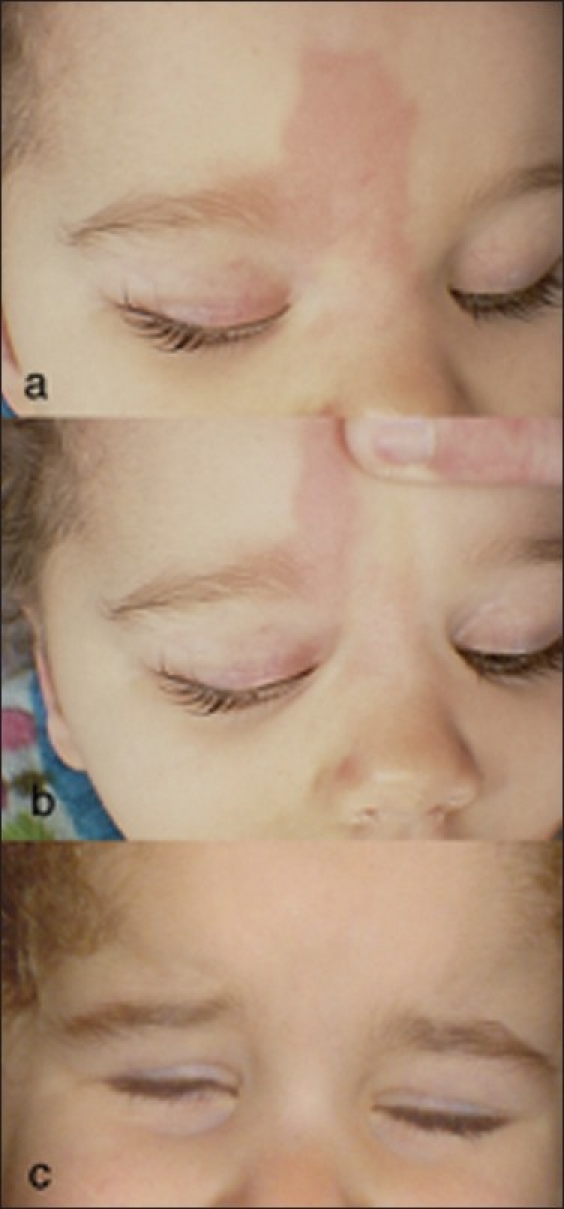
Hyperemic cutaneous periocular abnormality following intra-arterial chemotherapy for retinoblastoma. (a) Note the geographic erythematous plaque in the cutaneous area supplied by the supratrochlear and medial palpebral artery, also note the loss of the upper medial eyelashes. (b) See that the skin blanches with pressure. (c) Same patient 3 months following her last intra-arterial treatment. Note the resolution of the skin changes and eyelash loss

Of the 69 consecutive eyes of 63 children, 11 (16%) were found to have a hyperemic cutaneous periocular abnormality found in the skin. This condition occurred twice in one patient of the 12 occurences in 196 (6%) consecutive procedures. The redness consisted of a geographic erythematous plaque that blanched with pressure [Figure 2a, b]. The extent of the lesions corresponded to the distribution of the supratrochlear and medial palpebral arteries. The hyperemic cutaneous periocular abnormality was not present immediately after the infusion and was noted to appear within the first 24-48 hrs following the procedure, then faded slowly. No child had bilateral findings during tandem treatments. No intervention was given to the patients, and they had no symptoms of pain or pruritus. As it resolved spontaneously, no biopsy was recommended. The age of affected patients ranged from 3 to 120 months. All children’s eyes with the hyperemic cutaneous periocular abnormality received melphalan in doses ranging from 3 to 7.5 mg. Topotecan was used in two patients, and the dose ranged from 0.4 to 3.0 mg [Table 1]. It was noted during the intra-arterial infusion that in 4 (36%) out of the 11 patients, injection was made with the catheter in “wedge flow.” Wedge flow is characterized by a slowing of contrast distal to the catheter tip angiographically that occurs when the catheter tip partially obstructs the ophthalmic artery limiting normal arterial flow. This is in contrast to the 3/58 (5%) eyes that were noted to be in wedge flow that did not develop the cutaneous findings, two of the three eyes without cutaneous findings had mild conjunctival redness and swelling. No other complications were noted in children with the hyperemic cutaneous periocular abnormality except for eyelash loss. Eight out of the 11 (73%) eyes with the hyperemic cutaneous periocular abnormality also had associated eyelash loss on the medial third of the upper eyelid. Eyelash loss was always associated with skin changes and was not noted alone in the 58 eyes without skin erythema. The hyperemic cutaneous periocular abnormality and eyelash loss spontaneously resolved within 3 months following completion of the intra-arterial chemotherapy [Figure 2c].

**Table 1:** Patient demographic and procedure data in patients with hyperemic cutaneous periocular abnormal following intraarterial chemotherapy for retinoblastoma

Patient no.	Age, months	Drug used	Dose	Cycle seen	Comments
1	59	Mel./Topo.	5mg/1.5mg	3,5	-
2	12	Mel.	5mg	3	-
3	26	Mel.	7.5 mg	3	Wedge flow
4	6	Mel.	3 mg	1	-
5	9	Mel. /Topo.	6 mg/0.4mg	2	-
6	32	Mel.	5 mg	1	-
7	10	Mel.	4 mg	2	Wedge flow
8	120	Mel.	6 mg	2	-
9	6	Mel.	3	mg 1	-
10	34	Mel.	7.5 mg	3	Wedge flow
11	30	Mel./Carbo.	7.5 mg /3.0mg	3	Wedge flow

Mel. = Melphalan, Topo. = Topotecan, Carbo. = Carboplatin

## DISCUSSION

Reversible cutaneous hyperemia following intra-arterial chemotherapy has been described with nonophthalmic cancers. In treatment of advanced head and neck cancer with intra-arterial chemotherapy using vincristine, bleomycin, and methotrexate or cisplatin and bleomycin, local cutaneous reactions were described in 8% of cases of which nearly all spontaneously resolved.^4^ Melphalan itself has been associated with reticulate scleroderma and ulceration after isolated limb perfusion (ILP) for treatment of melanoma.^5^ In one study, erythema and localized swelling were described in 80% of 181 patients who underwent melphalan ILP for limb melanoma.^6^

Sixteen percent of our patients had a distinctive hyperemic cutaneous periocular abnormality following intra-arterial chemotherapy Intensity, size, and duration of the hyperemic cutaneous periocular abnormality varied among the 11 patients. Age, timing, type of chemotherapy used, and dose varied within the group. With regards to dose, patients with a reaction tended to be in the higher dose range for their age. During the procedure, four of 11 patients (36%) were in wedge flow. Wedge flow results in a less-diluted infusion of drug secondary to the partial obstruction of the ophthalmic artery by the catheter tip during the infusion. It is possible that this may contribute to the cutaneous changes observed in our patients. Seventy-three percentage of patients with the hyperemic cutaneous periocular abnormality also had ciliary madarosis. The resolution of the hyperemic cutaneous periocular abnormality preceded the re-growth of cilia in all cases. This side effect occurs in a small percentage of patients and is self-limited. It is unknown if the infusion technique, dose, or vascular anatomy contribute to its frequency.
